# Evidence-based appraisal of situational judgement tests (revisited)

**DOI:** 10.1016/j.clinme.2024.100020

**Published:** 2024-01-28

**Authors:** Gurvinder Sahota, John McLachlan, Fiona Patterson, Paul Tiffin

**Affiliations:** a University of Nottingham, Nottingham, United Kingdom; b University of Central Lancashire, Preston, United Kingdom; c Work Psychology Group, Derby, UK and City University of London, London, United Kingdom; d Hull York Medical School, University of York, York, United Kingdom

We were surprised that the letter by Sam *et al.*^1^ commenting on our opinion article^2^ responding to their own opinion article^3^ primarily mounted a defence of the shift to preference informed allocation, since we ourselves described this as having positive potential.

Instead, we had focused on correcting their misrepresentation of evidence for the predictive validity, issues relating to fairness, and other psychometric properties, of situational judgement tests (SJT) used in this context and in many other settings. Workforce policy interventions must be evidence-based and it is essential that relevant research is appraised and presented in a scientific and balanced manner.

Regarding validity, for example, Sam *et al.*^4^ state that ‘the large sample size meant the study had acceptable power despite the overall risk of disciplinary action being low’ – namely the dataset included only 65 doctors with this outcome. We have since conducted our own multivariate power analysis, using the R package `powerSurvEpi', based on information in the original report by Sam *et al.* We assumed that normalised Educational Performance Measure (EPM) and SJT scores correlate with a magnitude of around 0.3.^5^ This post-hoc power calculation indicated that the study actually only had around a 27 % probability of showing that the estimated adjusted HR of 0.84 (which we would consider substantively meaningful) was statistically significant at the p<0.05 level. Thus, the study was certainly underpowered to show a meaningful effect of the F1 SJT scores, adjusted for the EPMs. Thus, our assertion that the abstract of their original paper is misleading is supported.

Regarding fairness, we don't dispute the presence of F1 SJT score differences between Black and Minority Ethnic (BAME) and White students, as is evident for almost all assessments elsewhere, but we dispute that this equates to the test being `biased'^3^ without a more sophisticated causal explanation of the issues, which Sam *et al.* themselves now admit is `unclear'.^1^

SJT scores are normally distributed; therefore most candidates have scores in the middle of the distribution, with small differences between them. Inevitably, small score changes there lead to larger changes in ranked position. To make the fine differentiations required to rank 8,000 candidates exactly would require unattainable levels of accuracy for any assessment.

With regard to the use of the SEM, Cronbach's α for the SJT is high (0.83–0.86 in 2023), exceeding the requirement for a high-stakes test. Since the standard deviation is appropriate for the distribution, the SEM is therefore comparable with any test with these properties, and consequently the reliability is good.

Similarly, the authors’ argument regarding everyone being awarded 41 points is meaningless – the key issue here is the variance. As stated in our previous response, the SJT and EPM have been scaled so that when combined, the variance of each score determines the weighting.

Regarding the concordance analysis using Kendall's W, this is a relatively small, initial step of developing the scoring key, and in practice, given that 0.5 is the minimum acceptable value the vast majority of the items have considerably larger values. The scoring key is actually determined by psychometric analyses of large-scale pilot data with candidates.

Similarly, Sam *et al.*^1^ question the robustness and fairness of the F1 SJT where they repeat the canard about the SJT being a `randomiser'. Why then did Brown *et al.*,^6^ in their analysis of differential attainment by medical school attended, use a combination of the EPM and SJT as the primary outcome measure, to judge both differential attainment and the size of the awarding gaps by medical school attended?

Our discussions regarding why differential attainment occurred in the SJT were avoided in the authors' response.^1^ It is highly likely that a similar, possibly higher, level of differential attainment for outcomes will be observed in the forthcoming Medical Licensing Assessment (MLA) and an adequate explanation of any sub-groups' differences will be essential. Or will this assessment be deemed unacceptable if differential attainment is demonstrated?

Sam *et al.*^1^ state that `determination of graduate placements has never been an issue of personnel selection – it is one of allocation'. The vast majority of students are indeed allocated a place but crucially, the implications of the F1 SJT being removed from the process also needs to be appreciated and understood. For example, over several years a small, but important, number of students score extremely poorly on the SJT, which identifies potential competency issues and readiness to enter Foundation training. It would be wise to retain the use of an SJT or a similarly reliable measure of professional attributes, perhaps for formative purposes before students graduate, where early identification of such issues could be possibly remediated.
